# The Establishment of the Pfizer-Canine Comparative Oncology and Genomics Consortium Biospecimen Repository

**DOI:** 10.3390/vetsci2030127

**Published:** 2015-07-07

**Authors:** Christina Mazcko, Rachael Thomas

**Affiliations:** 1 Comparative Oncology Program, Center for Cancer Research, National Cancer Institute, Bethesda, MD 20892, USA; 2 Department of Molecular Biomedical Sciences, College of Veterinary Medicine, North Carolina State University, Raleigh, NC 27607, USA; E-Mail: rthomas3@ncsu.edu; 3 enter for Comparative Medicine and Translational Research, North Carolina State University, Raleigh, NC 27607, USA

The Canine Comparative Oncology and Genomics Consortium (CCOGC) was formed in 2004 in an effort to capitalize on the generation of a domestic dog genome sequence assembly [1], which created new opportunities to investigate canine cancers at the molecular level [2]. At the time of the CCOGC inception, the most critical resource needed to achieve this goal was a comprehensive and well-annotated repository of clinical specimens from dogs with naturally occurring cancer. The CCOGC defined as a primary objective the establishment of a biorepository of clinical samples from 3,000 dogs with commonly diagnosed cancers that are also associated with broad comparative and translational relevance. Seven histologies were selected to populate the bank, with the aim of recruiting 600 cases each of osteosarcoma, lymphoma, and melanoma, and 300 cases each of hemangiosarcoma, soft tissue sarcoma, mast cell tumor, and pulmonary tumor.

The start-up costs to develop and maintain a repository of specimens from 3,000 dogs was estimated to be $2.1 million. This included the physical infrastructure, database development and management, and collection and maintenance of clinical specimens with assurance of stable sample quality during long-term storage. A lead gift of $1.1 million was received from Pfizer, and with it Pfizer received the naming rights to the biospecimen repository. Additional substantial donations were received from the Morris Animal Foundation and the American Kennel Club Canine Health Foundation. The National Cancer Institute (NCI) provided support for the physical infrastructure for the Pfizer-CCOGC biospecimen repository, which is housed at Fisher Bioservices in Frederick, Maryland. In 2007, the CCOGC was incorporated as a not for profit entity with 501c3 status. Two sustainable revenue streams were defined, the first being requests for samples from the research community following a predefined cost structure. The second proposed revenue stream was through targeted recruitment of prospective specimens on behalf of individual investigators, using the established CCOGC infrastructure.

Standard operating procedures (SOPs) for sample collection were established by the CCOGC Bank Committee, which is composed of key leaders in the field of comparative oncology and representatives from each collecting institution. A universal SOP was implemented that applied to each of the seven histologies and outlined the collection of all required fluids (serum, whole blood, plasma and urine), along with requirements for specimen labeling and shipping. Histology-specific SOPs were attached as appendices. These appendices included specific inclusion and exclusion criteria along with requirements for collection of representative tumor tissue, in addition to matched normal (non-neoplastic) tissue from the same case. Each submission required the provision of both formalin-fixed and OCT-fixed tumor and normal tissue samples, in addition to non-fixed specimens snap-frozen in liquid nitrogen. Tissue and blood samples were to be partitioned into multiple representative portions at the collection site, prior to transfer to the biorepository, for maintenance of sample integrity and to maximize their downstream utility. There were no restrictions placed on the breed, age or sex of the dog. All samples were to be collected from client-owned dogs prior to initiation of therapy and with informed owner consent.

In 2006 the CCOGC issued an initial request for proposals from potential sample collection sites, which was circulated to veterinary academic institutions in the mainland USA. Essential criteria included having dedicated medical and surgical oncology services, pathology services with 52-week coverage, experience with tissue archiving and clinical trials, and a veterinary clinical caseload appropriate for achieving target collection numbers. Three sites (Colorado State University, The Ohio State University, and University of Wisconsin-Madison) were selected in 2006 to pilot the SOPs for sample collection. Four additional sites (Michigan State University, Tufts University, University of California-Davis, and University of Missouri) were added in 2007 and an eighth collection site (University of Tennessee) joined in 2009. The SOPs were approved by each Institutional Animal Care and Use Committee (IACUC).

To date, the biospecimen repository has received over 60,000 specimens from almost 2,000 dogs. These include samples from 511 osteosarcomas, 446 lymphomas, 329 soft tissue sarcomas, 186 mast cell tumors, 184 hemangiosarcomas, 108 melanomas, and 100 pulmonary tumor cases. One hundred breeds are represented in the biospecimen repository, with golden retrievers (13%), Labrador retrievers (13%) and mixed breed dogs (22%) being highly represented. Patient age at time of collection ranged from <1 to 20 years with a majority being collected between at 7–10 years of age (51%). Patient demographics, pathology reports, treatment information, and clinical follow-up every six months (until time of death or a patient is lost to follow up) have been recorded for all cases. A web-based system, CCOGC DogBank Tissue Tracker, was developed by the NCI to enable entry of patient information at the collection sites. Queries of the biorepository inventory are made through Labmatrix, a CaBig-compliant web-accessible clinical and biological relational database that permits the application of dozens of filters to optimize sample selection.

In 2012, an internally commissioned quality assessment of the Pfizer-CCOGC biospecimen repository was performed, prior to the release of specimens to the research community. Quality control and quality assurance parameters were assessed on a panel of biospecimens distributed randomly across the eight submitting institutions and the seven tumor histologies represented in the repository. The first phase consisted of a comprehensive histopathologic review of formalin-fixed and paraffin-embedded (FFPE) specimens from 331 tumors, representing 40–50 cases of each histology. A panel of board-certified veterinary pathologists from the NCI examined hematoxylin and eosin (H&E)-stained sections from each tumor, assigning a diagnostic classification that was subsequently compared with that associated with the original case submission. The proportion of cases for which the FFPE specimen examined at the NCI corroborated the original diagnosis ranged from 64% (hemangiosarcoma) to 100% (lymphoma and osteosarcoma), with a mean of 89% across the total of 331 cases. An alternative diagnosis was reached for 6% of all specimens, and the remaining 5% of specimens showed no conclusive evidence for the presence of a neoplastic process. The low value for hemangiosarcoma was due primarily to lack of sufficient tumor tissue in the submitted specimen to permit conclusive diagnosis. Hemangiosarcoma is associated with particularly extensive cellular, anatomical and clinical heterogeneity, which correlates with broad molecular variation both within and between tumors, and marked attenuation of tumor-associated genomic abnormalities by non-neoplastic cells [3]. The ability to quantify tumor heterogeneity is therefore becoming increasingly critical as molecular studies increase in scope, validity and applicability.

To this end, tumor heterogeneity was assessed in 132 cases from the three major histologies (osteosarcoma, lymphoma, and melanoma) through identification of stromal contamination in FFPE sections using routine histopathology, immunohistochemistry and a novel quantitative morphometry technique [4]. The vast majority of lymphoma cases exhibited a high proportion of tumor cells (median 94%), while the values for melanoma (median 84%) showed a broader range. In contrast, osteosarcomas showed extensive variation, with most cases containing <50% tumor (median 39%). The presence of normal stroma in tumor specimens may confound downstream studies of tumor DNA, RNA and protein by diluting out neoplastic signatures and rare mutation events. It is therefore critical that intratumoral heterogeneity is a major consideration during experimental design, sample selection and data interpretation.

The second component of the biospecimen repository assessment was carried out to assess the quality and yield of nucleic acid obtained from fresh-frozen tumor tissue and whole blood, since these represent the most commonly utilized reagents destined for downstream molecular studies. A total of 188 cases were selected at random from the biospecimen repository, providing proportional representation of each tumor histology and submitting institution. Routine techniques were used to extract total genomic DNA from tumor and blood specimens, and total RNA was isolated from tumor tissue. Certain histology-associated limitations were noted, such as reduced yields among hemangiosarcomas due to low cellularity and the presence of necrotic tissue, and in osteosarcoma due to challenges with effective tissue disruption. Pigmentation associated with melanotic lesions also impacted sample purity in a small proportion of melanomas. Overall, however, the quality scores for the majority of specimens fell in the highest category for nucleic acid yield and integrity (73% of tumor DNAs, 64% of tumor RNAs and 81% of blood RNAs), readily exceeding standard criteria for application in current molecular biology techniques. Conclusions from the quality assessment of the Pfizer-CCOGC biospecimen repository were presented at the 2013 American Association for Cancer Research Special Conference on the Translational Impact of Model Organisms on Cancer [5].

In October 2013, after successful completion of the quality assessment phase, the CCOGC opened the biospecimen repository to the research community. A price structure was implemented to allow for the replenishment of the repository as samples are released. To date, the Pfizer-CCOGC biospecimen repository has released over 1,900 samples and completed one prospective collection. In an effort to maximize the future utility of the repository, the CCOGC recently eliminated the requirement for scientific review and project disclosure. Investigators apply online and include the number and nature of samples requested, along with any criteria required for additional sample filtering. Samples matching these criteria are then identified within the specimen inventory and a request is made to the repository for those samples to be retrieved and shipped to the investigator. The average turnaround time from completion of sample request to shipment is 2–3 weeks.

Through generous donations and the efforts of the CCOGC officers and collection sites the Pfizer-CCOGC Biospecimen Repository has become one of the most valuable resources in the field of comparative oncology. The CCOGC has laid the groundwork for biobanking in the comparative oncology community, and the comprehensive quality assurance evaluation of the repository reinforces its tremendous potential to advance canine and comparative cancer studies. Indeed, this effort has spawned additional ongoing biobanking efforts at both CCOGC-participating and non-participating institutions.

Additional information about the CCOGC can be found at www.ccogc.org.

## References

[1] Lindblad-Toh K., Wade C.M., Mikkelsen T.S., Karlsson E.K., Jaffe D.B., Kamal M., Clamp M., Chang J.L., Kulbokas E.J., Zody M.C. (2005). Genome sequence, comparative analysis and haplotype structure of the domestic dog. Nature.

[2] Khanna C., Lindblad-Toh K., Vail D., London C., Bergman P., Barber L., Breen M., Kitchell B., McNeil E., Modiano J.F. (2006). The dog as a cancer model. Nat. Biotechnol..

[3] Thomas R., Borst L., Rotroff D., Motsinger-Reif A., Lindblad-Toh K., Modiano J.F., Breen M. (2014). Genomic profiling reveals extensive heterogeneity in somatic DNA copy number aberrations of canine hemangiosarcoma. Chromosome Res..

[4] Webster J.D., Simpson E.R., Michalowski A.M., Hoover S.B., Simpson R.M. (2011). Quantifying histological features of cancer biospecimens for biobanking quality assurance using automated morphometric pattern recognition image analysis algorithms. J. Biomol. Tech..

[5] Thomas R., Simpson M., Mochizuk I.H., Williams C., Poorman K., Kennedy K., Mazcko C., Modiano J., Breen M. Quality control and quality assurance of canine biological specimens available through the Pfizer-CCOGC biospecimen repository for comparative oncology studies. Proceedings of American Association for Cancer Research Special Conference: Translational Impact of Model Organisms on Cancer.

