# Addendum: Mazcko, C., *et al*. The Establishment of the Pfizer-Canine Comparative Oncology and Genomics Consortium Biospecimen Repository. *Vet. Sci.* 2015, *2*, 127–130

**DOI:** 10.3390/vetsci2040406

**Published:** 2015-11-06

**Authors:** Christina Mazcko, Rachael Thomas

**Affiliations:** 1 Comparative Oncology Program, Center for Cancer Research, National Cancer Institute, Bethesda, MD 20892, USA; 2 Department of Molecular Biomedical Sciences, College of Veterinary Medicine, North Carolina State University, Raleigh, NC 27607, USA; E-Mail: rthomas3@ncsu.edu; 3 Center for Comparative Medicine and Translational Research, North Carolina State University, Raleigh, NC 27607, USA

The authors wish to make the following correction to their paper published in *Veterinary Sciences* [1]: Please add the Acknowledgements Section with the following:

**Acknowledgements:** This research was supported in part by the Intramural Research Program of the National Institutes of Health (NIH), NCI, Center for Cancer Research. The results and interpretations presented herein do not reflect the views of the US Government. 

The authors would like to apologize for any inconvenience caused to readers by this change.
